# Progeroid laminopathy with restrictive dermopathy-like features caused by an isodisomic LMNA mutation p.R435C

**DOI:** 10.18632/aging.100566

**Published:** 2013-06-19

**Authors:** Sven Starke, Peter Meinke, Daria Camozzi, Elisabetta Mattioli, Roland Pfaeffle, Manuela Siekmeyer, Wolfgang Hirsch, Lars Christian Horn, Uwe Paasch, Diana Mitter, Giovanna Lattanzi, Manfred Wehnert, Wieland Kiess

**Affiliations:** ^1^ Department of Women and Child Health, Hospital for Children and Adolescents, Centre of Pediatric Research, University Hospital, University of Leipzig, Leipzig, Germany; ^2^ Department of Pediatric Oncology, Hematology and Hemostaseology, Hospital for Children and Adolescents, University Hospital, University of Leipzig, Leipzig, Germany; ^3^ Institute of Human Genetics Greifswald, University Medicine Greifswald and Interfaculty, Institute of Genetics and Functional Genomics, University of Greifswald, Germany; ^4^ National Research Council of Italy, CNR, Institute of Molecular Genetics, Unit of Bologna, Italy; ^5^ Department of Pediatric Radiology, University Hospital of Leipzig, Leipzig, Germany; ^6^ Institute of Pathology, Division of Breast, Gynecologic and Perinatal Pathology, University of Leipzig, Leipzig, Germany; ^7^ Department of Dermatology, Venereology, and Allergology, University Hospital of Leipzig, Leipzig, Germany; ^8^ Institute of Human Genetics, University Hospital of Leipzig, Leipzig, Germany

## Abstract

The clinical course of a female patient affected by a progeroid syndrome with Restrictive Dermopathy (RD)-like features was followed up. Besides missing hairiness, stagnating weight and growth, RD-like features including progressive skin swelling and solidification, acrocontractures, osteolysis and muscular hypotension were observed until the patient died at the age of 11 months. A homozygous *LMNA* mutation c.1303C>T (p.R435C) was found by Sanger sequencing. Haplotyping revealed a partial uniparental disomy of chromosome 1 (1q21.3 to 1q23.1) including the *LMNA* gene. In contrast to reported RD patients with *LMNA* mutations, LMNA p.R435C is not located at the cleavage site necessary for processing of prelamin A by ZMPSTE24 and leads to a distinct phenotype combining clinical features of Restrictive Dermopathy, Mandibuloacral Dysplasia and Hutchinson-Gilford Progeria. Functionally, LMNA p.R435C is associated with increasing DNA double strand breaks and decreased recruitment of P53 binding protein 1 (53BP1) to DNA-damage sites indicating delayed DNA repair. The follow-up of the complete clinical course in the patient combined with functional studies showed for the first time that a progressive loss of lamin A rather than abnormal accumulation of prelamin A species could be a pathophysiological mechanism in progeroid laminopathies, which leads to DNA repair deficiency accompanied by advancing tissue degeneration.

## INTRODUCTION

Laminopathies are a group of rare genetic diseases caused by mutations in genes encoding proteins of the nuclear lamina (primary laminopathies) or proteins interacting with lamina proteins (secondary laminopathies). A group of these laminopathies have been classified as progeroid syndromes that can be caused by mutations in *LMNA* or *ZMPSTE24* [1]. *LMNA* is encoding lamin A and lamin C by alternative splicing, while *ZMPSTE24* is encoding the zinc metalloproteinase ZMPSTE24, which is necessary for the processing of prelamin A to mature lamin A. Therefore *LMNA* mutations are classified as primary laminopathies whereas *ZMPSTE24* mutations are included in the group of the secondary laminopathies. Progeroid syndromes mimic clinical and molecular features of aging. Apart from some atypical progeroid forms there are three major syndromes caused by *LMNA* or *ZMPSTE24* mutations: Hutchinson Gilford progeria syndrome (HGPS), Mandibuloacral Dysplasia (MAD) and Restrictive Dermopathy (RD).

HGPS is most commonly caused by the heterozygous *de novo* lamin A mutation p.G608G [2-4], which activates a cryptic splice site and causes a deletion of 50 amino acids on the protein level including the C-terminal cleavage site for ZMPSTE24. The resulting farnesylated mutant lamin A, known as progerin, accumulates inside the nucleus. The affected children appear healthy at birth and, in the course of 1-2 years progressively develop a so-called progeroid phenotype, comprising extreme short stature, low body weight, early loss of hair, lipodystrophy, scleroderma, decreased joint mobility, osteolysis, and facial features resembling aging[2, 5]. Cardiovascular problems lead in most cases to death in the second decade.

MAD can be associated with either homozygous or compound heterozygous missense mutations in *LMNA* (MADA) or a combination of a nonsense and a missense mutation in *ZMPSTE24* (MADB) [6-8]. MAD patients are characterized by postnatal growth retardation, craniofacial anomalies like mandibular hypoplasia (or osteolysis) and protruding mid-face as well as skeletal anomalies including progressive osteolysis of the terminal phalanges and clavicles. Skin changes like atrophy and speckled hyperpigmentation have been observed. Furthermore clinical features like displaced teeth, thin and brittle hair, short and wide fingernails as well as accumulation of fat in the neck and moderate lipodystrophy of the limbs are typical.

RD describes phenotypically a lethal neonatal genodermatosis characterized by tautness of the skin causing fetal akinesia and often premature delivery. Other clinical features include tightly adherent thin skin, prominent vessels, characteristic facial features, generalized joint contractures, dysplasia of clavicles and respiratory insufficiency. The clinical course of RD is fatal and, with respect to HGPS or MAD, more severe leading to neonatal death or death in early infancy [9]. *LMNA*-linked heterozygous RD mutations cause a skipping of exon 11 and lead to a deletion of 90 amino acids [7]. The resulting truncated protein cannot undergo full posttranslational maturation, similar to progerin, and is accumulated inside the nucleus. In *ZMPSTE24*-linked RD, homozygous or compound heterozygous nonsense mutations are causing the complete absence of the essential prelamin A processing enzyme ZMPSTE24 resulting in an extreme accumulation of normal length lamin A precursors.

A common feature of at least HGPS and RD is the accumulation of prelamin A or truncated forms of it. Pathogenic effects of prelamin A accumulation have been associated with altered chromatin organization and impaired DNA-damage response pathways, resulting in increased genomic instability, susceptibility to DNA-damaging agents and double strand breaks (dsb). Indeed such abnormalities are observed in patients with HGPS or RD as well as in cultured human and murine cells with aberrant lamin A processing pathways [10, 11]. On the other hand, increased genomic instability has been also reported in *LMNA*-knockout cells completely lacking lamin A [12].

Here we present a distinct primary progeroid lamino-pathy with overlapping features of HGPS, MAD and RD that is pathophysiologically not related to the accumulation of abnormally processed lamin A species, but rather is characterized by a loss of lamin A resulting in accumulation of dsb and impaired DNA repair.

## RESULTS

### Clinical Course

**Figure 1:**
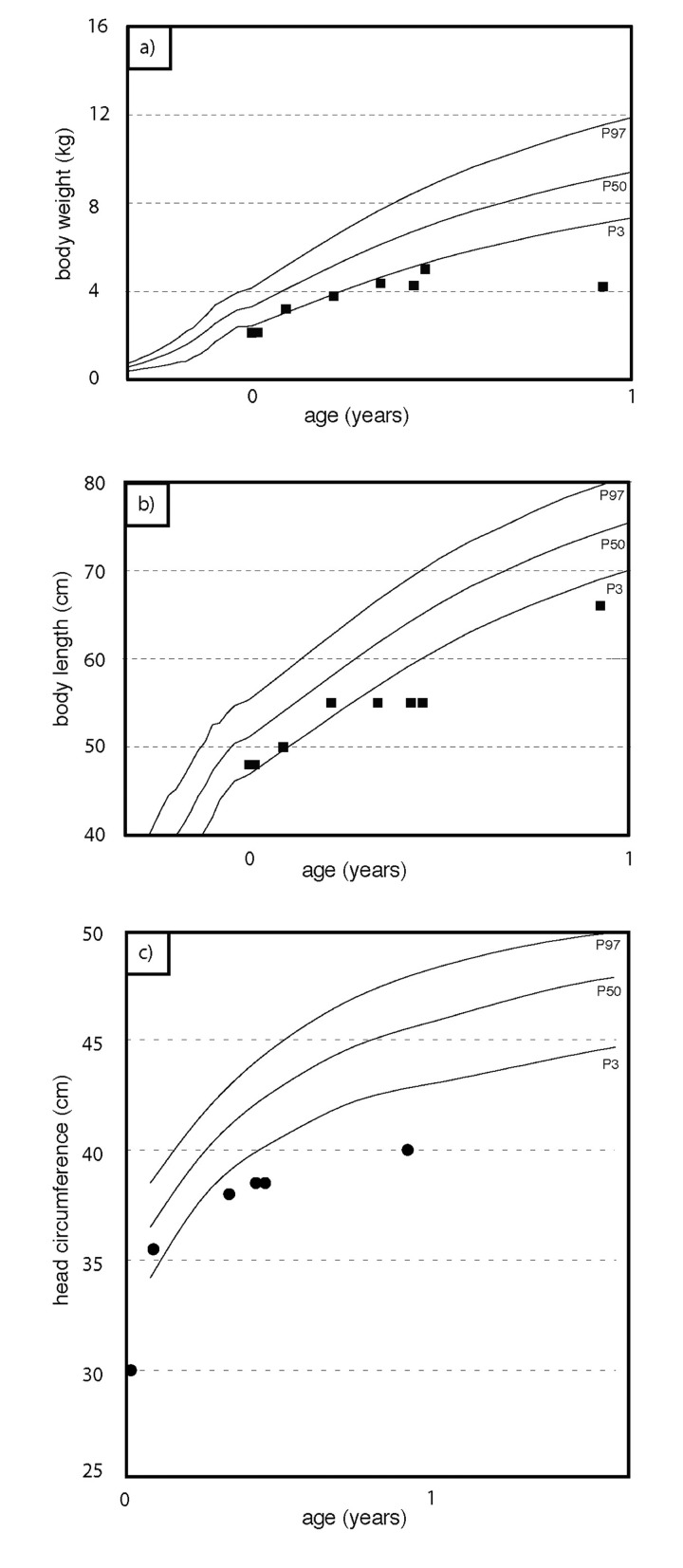
Weight percentiles (**a**), growth percentiles (**b**), head circumference percentiles (**c**) during the course of the disease. At birth, the patient was small for gestational age. Weight gain stagnates at about two months of age. Length and head circumference growth rank below 3^rd^ percentile.

**Figure 2:**
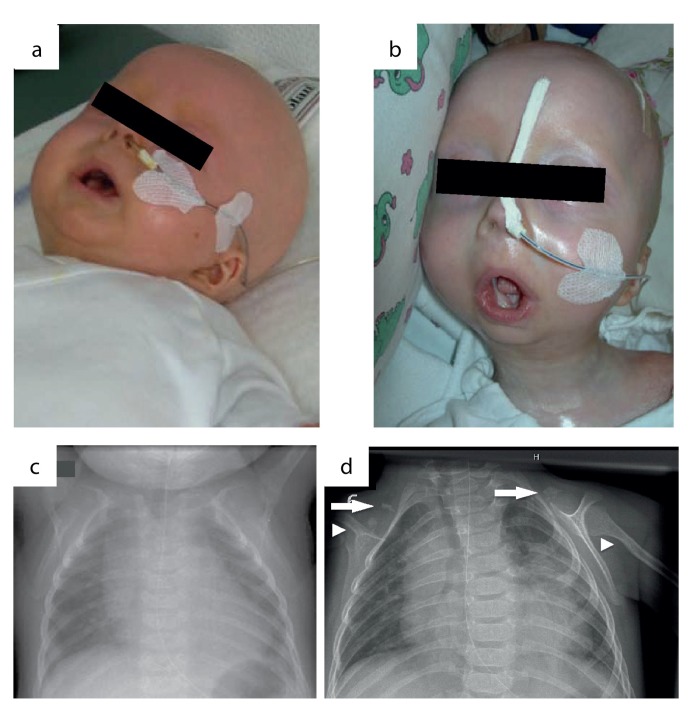
Phenotypic changes in the course of disease progression at age of 4 months (a) and age of 11 months (b). Chest x-rays were performed at age of 4 (c) and 11 months (d). Normal skeletal findings at age of 4 months are displayed. Missing of both claviculae at age of 11 months, residual bone fragments are denoted by arrows. Deformation and narrowing of both humeri at age of 11 months are marked by arrowheads.

**Figure 3:**
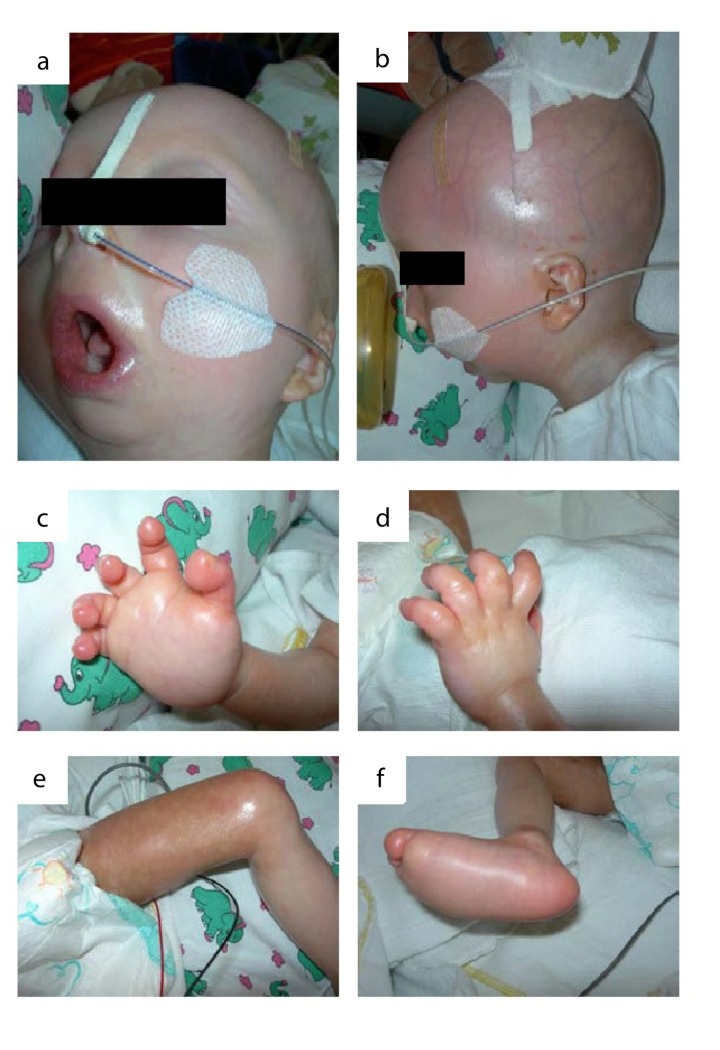
The patient at the age of 11 months: (**a**) microstomia, fixed in an o-shaped position (**b**) prominent superficial scalp veins, dysplastic auricels, microretrognathia. (**c-e**) acrocontractures. (**f**)„rocker bottom“ foot with prominent calcaneus and rounded bottom.

We report on an infant girl born at term by caesarian section due to nuchal cord after normal course of pregnancy. At newborn age, she was small for gestational age (birth weight below 3^rd^ percentile, birth length below 10^th^ percentile, head circumference below 3^rd^ percentile). She presented with muscular hypotension sucking weakness and slightly impaired swallowing. At the age of two months, skin turgors and a generalized failure to thrive were noticed. Weight gain had been stagnating since then on, length and head circumference growth remained below 3^rd^ percentile (Fig.1). At the age of four months, her skin appeared sclerotic and turgid with thereby initiating acrocontractures; the body fat and body hair were almost absent, and the child suffered from generalized hyperhidrosis, pruritus and touch sensitivity. As an attempt of treatment, a short course of steroids was given tentatively. However, the disease underwent continuous progression and syndromal features, including microstomia, microretrognathia, dysplastic auricles, small pinched nose, prominent superficial scalp veins, hands in claw-hand position and rocker bottom feet, were advancing (Fig.2a, b and 3).

During the course of the disease, the skin became generally inflexible and firm, apparently encasing the patient and resulting in mounting flexion contractures and defective positions of the joints. The miniaturized mouth could not be closed anymore, and was fixed in an o-shaped position. The respiratory chest movements were almost abolished by thoracic rigidity. Finally, at the age of 11 months, the patient acutely developed pneumonia and died as a result of global respiratory insufficiency. Strikingly at that point, chest x-rays that had been normal at the age of four months now revealed the absence of clavicles and a deformity of both humeri (Fig.2c, d). Except for slightly elevated erythrocyte sedimentation rate, white blood cell and platelet count, laboratory findings revealed normal results at all time. The histology of skin samples taken at the age of 11 months showed progressively flattened rete ridges and poorly developed skin appendages (Fig.5b).

### Mutational Analyses

The clinical data suggested a laminopathy. Thus, the complete coding regions including intron/exon boundaries of the *LMNA* (ENSG00000160789, ENST00000368300) and *FACE1* (ENSG00000084073, ENST00000372759) genes were PCR-amplified (Supplementary Material, Table S1) and used for direct Sanger sequencing. Although no changes in the *FACE1* gene were found, there were five homologous changes in the *LMNA* gene. Four of these five changes are known single nucleotide polymorphisms with frequencies of 15% or more (two intronic and two silent), while the base substitution c.1303C>T resulted in a pathogenic amino acid substitution (p.R435C). Interestingly, this previously described pathogenic mutation [13, 14] has been found with a low frequency of 0.023% in a Caucasian reference population (rs150840924, Supplementary Material, Fig.S1).

### Family analysis

**Figure 4:**
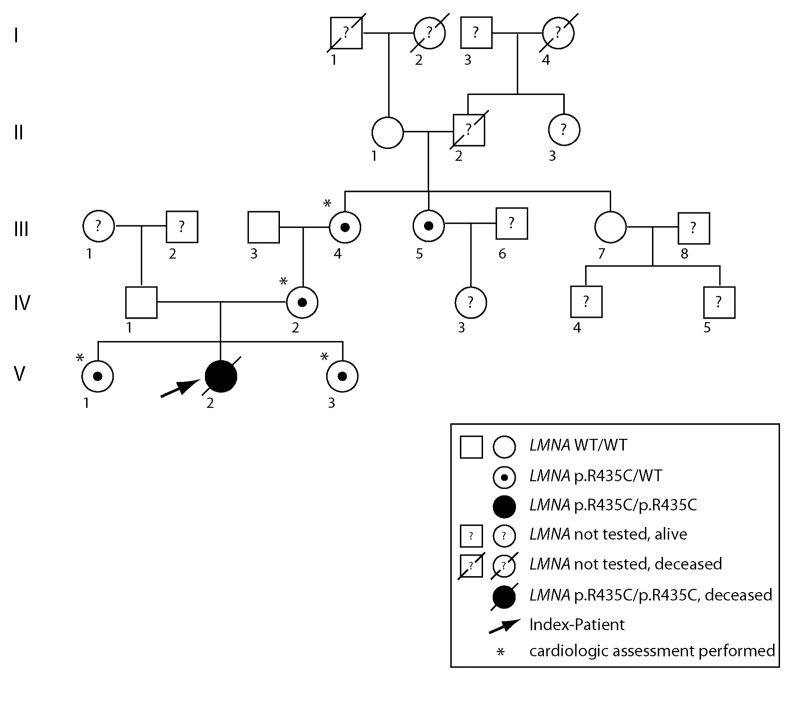
Pedigree of the affected family. Uniparental isodisomy of chromosome 1q21.3- q23.1 (involving the complete *LMNA* gene), causing homozygosity of autosomal recessively inherited *LMNA* mutation, was proven for the index patient. Except for the index patient, none of the displayed individuals showed signs of progeroid disease, or signs of progressive cardiac disease. Dilated cardiomyopathy was excluded by echocardiography in individuals labeled by *.

Sequencing of the *LMNA* gene in the patient's family showed that besides the mother, also the two sisters, the maternal grandmother, and the maternal grand aunt of the patient were heterozygous for p.R435C (Fig.4). All of them appeared to be healthy. Intriguingly, the patient's mother was heterozygous for the *LMNA* c.1303C>T (p.R435C) mutation, but the father was homozygous wild-type. Wrong paternity as an explanation of the patient's homozygosity was excluded. Multiplex Ligation-dependent Probe Amplification (MLPA) analysis to check for copy numbers showed two copies of the *LMNA* gene in the patient and thus excluded a deletion as a reason for the homozygous mutation (Supplementary Material, Fig.S2). Microsatellite marker analysis on chromosome 1 (Supplementary Material, Table S2) revealed a partial uniparental disomy of chromosome 1 (at least from 1q21.3 to 1q23.1) including the *LMNA* gene (Supplementary Material, Fig.S3), which would explain the homozygous *LMNA* c.1303C>T (p.R435C) mutation in the patient.

Echocardiographic assessment of the family members heterozygous for LMNA p.R435C mutation revealed the following results (see Fig.4):

Patient III-4 (age 61 years, body weight 67kg, body length 149cm), showed signs of (age related) hypertensive cardiac disease in terms of moderate dilatation of the left atrium and moderate concentric muscular hypertrophy of the normal sized left ventricle (LV). Left ventricular function was normal except for discrete inferior hypokinesia, with an ejection fraction (EF) of 57% and a fraction of shortening (FS) of 29%. The right ventricle (RV) was normal sized. Furthermore, discrete degenerative alterations of the aortic and mitral valve could be shown, but there was no evidence of inherent structural or functional cardiac abnormalities.

Patient IV-2 (age 40 years, body weight 59kg, body length 158cm) showed no signs of structural cardiac disease. LV and RV were normal sized. The functional tests revealed normal results (LV EF 58%, FS 32%). Except for discrete degenerative alteration of the aortic valve, there were no structural valvular alterations.

Patient V-1 (age 13years, body weight 45kg, body length 160cm) showed no signs of structural cardiac disease. LV and RV were normal sized. The functional tests revealed normal results (LV EF 72%, FS 40%). There were no signs of valvular alterations.

Patient V-3 (age 3 years, body weight 16 kg, body length 110cm) showed no signs of structural cardiac disease. LV and RV were normal sized. The functional tests revealed normal results (LV EF 63%, FS 33%). There were no signs of valvular alterations.

### Functional Analyses

**Figure 5:**
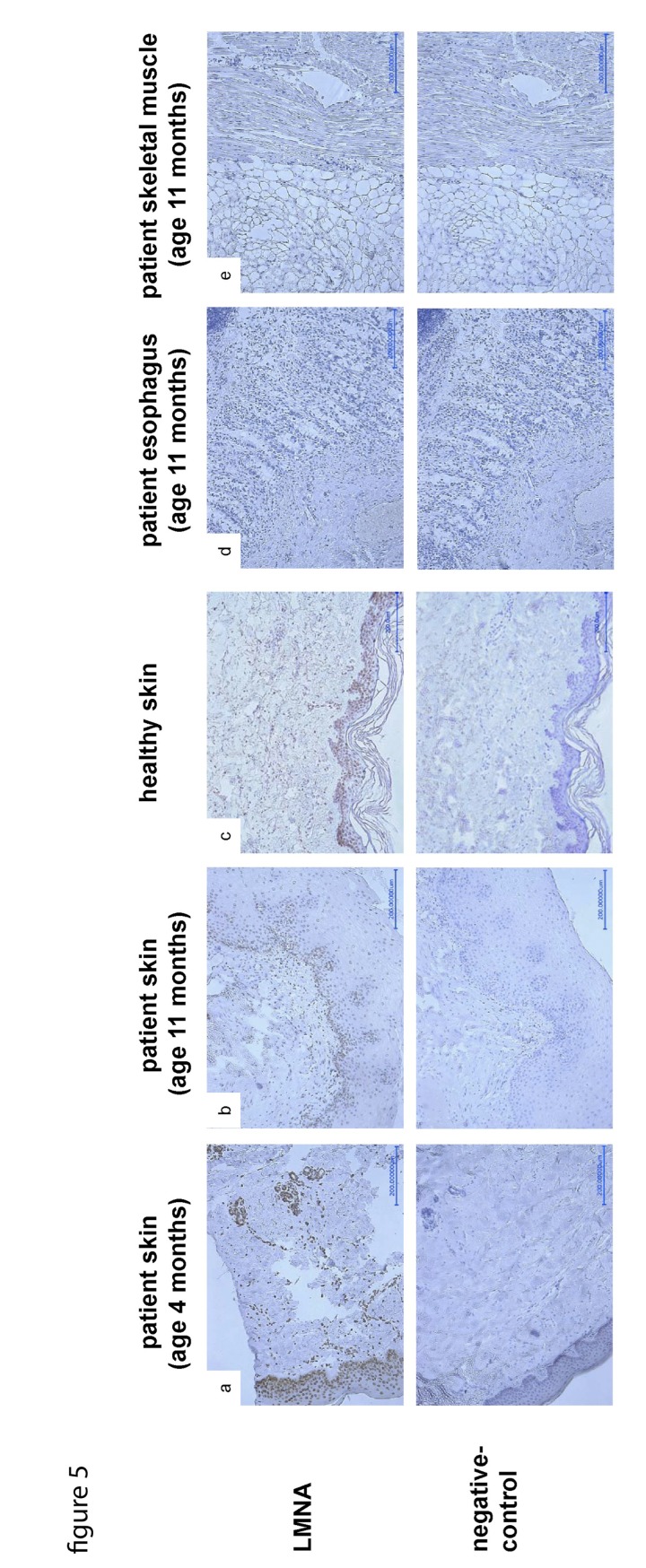
LMNA-staining of skin samples taken at age of 4 (**a**) and 11 (**b**) months respectively, shows decreasing amount and signal intensity of LNMA-positive nuclei in the dermo-epidermal junction zone compared to control skin (**c**). As a secondary finding, the rete ridges are progressively flattened, the skin appendages are poorly developed. LMNA-staining of samples taken from esophagus (**d**) and skeletal muscle (**e**) during autopsy does not detect any LMNA signal.

The LMNA p.R435C mutation is predicted not to be located at the cleavage site necessary for processing of prelamin A by ZMPSTE24. Prelamin A staining can be used as a marker for abnormal accumulation of unprocessed lamin A at the nuclear lamina. We found that only a few nuclei in our patient's skin were positive for prelamin A (Supplementary Material, Fig.S4) confirming a normal prelamin A processing. When we performed lamin A-staining of patient skin samples, we found decreasing numbers of lamin A-positive nuclei in the dermo-epidermal junction zone between the 4^th^ and 11^th^ month of life (Fig.5a, b). The signal intensity was also decreasing during this time interval. Additionally, staining of autopsy samples from esophagus and skeletal muscle did not detect any lamin A signal (Fig.5d, e).

**Figure 6:**
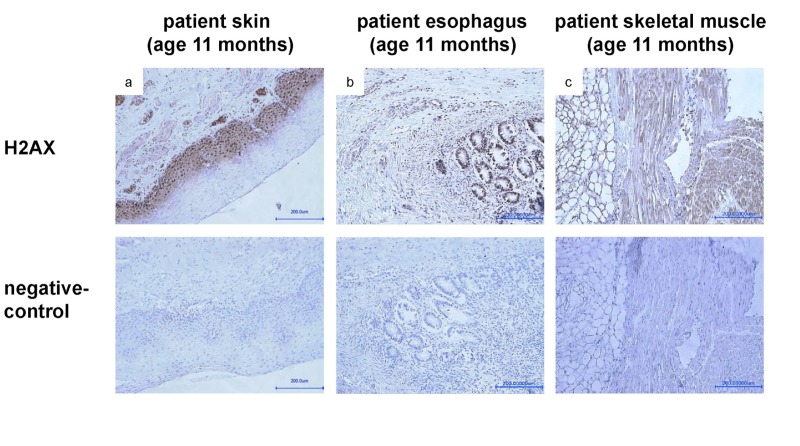
Staining with anti-gamma H2AX (phospho S139) antibody of samples taken from skin (**a**), esophagus (**b**) and skeletal muscle (c) during autopsy at age of 11 months shows a strong signal, indicating presence of DNA double strand breaks.

To probe for the presence of DNA dsb, staining with gamma H2AX antibody was done. The staining in different autopsied tissues at the age of 11 months revealed a strong immuno-reactivity consistent with the presence of significant DNA dsb (Fig.6a-c). For further functional analyses, we performed a heterologous expression of either recombinant mCherry-tagged *LMNA* p.R435C or mCherry-tagged wt *LMNA* in HEK-293 cells. The localization of the mutant protein at the nuclear envelope was similar to that of wild type, although some p.R435C lamin A aggregates were observed (not shown). This strongly suggests that despite the mutation, LMNA p.R435C is able to assemble in its normal region beneath the nuclear membrane.

**Figure 7:**
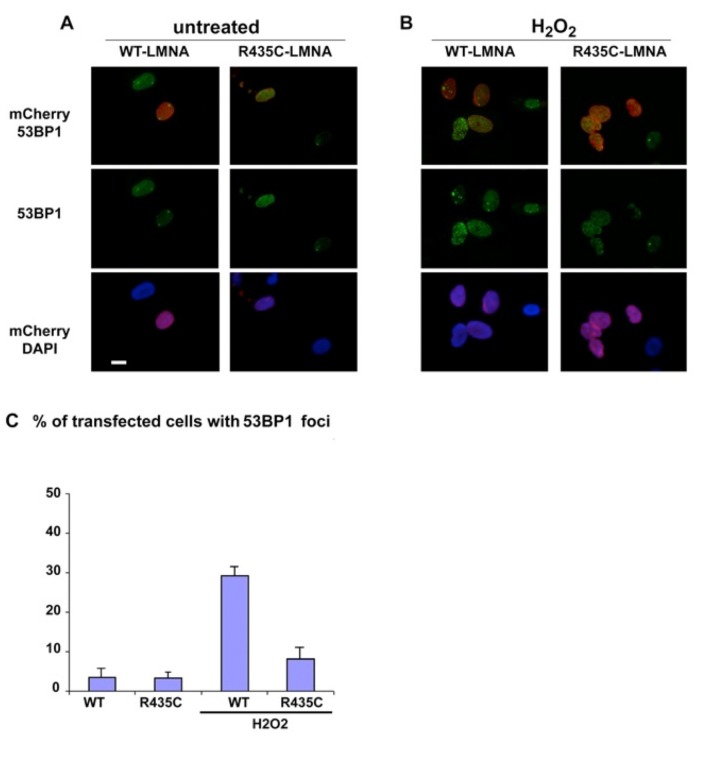
53BP1 localization in cultured human normal fibroblasts transfected with either mCherry-tagged *LMNA*-p.R435C or mCherry-tagged wt- *LMNA* plasmids. (**A**) untreated or (**B**) treated with hydrogen peroxide (H_2_O_2_). 53BP1 was specifically labeled using a monoclonal antibody and revealed by FITC –conjugated anti-mouse IgG secondary antibody (green). (**C**) Quantitation of 53BP1 foci in H_2_O_2_ –treated cells. Means of three different counts performed in separate experiments are reported +/− standard deviation. The difference is statistically significant (p<0.05 by the Student's T-test).

Since the staining with anti-gamma H2AX indicated significant DNA dsb in the patient's autopsy, we tested the response of 53BP1 protein following oxidative stress in a cultured cell model containing the recombinant mutant lamin A. Human normal fibroblasts were transfected with either tagged *LMNA* p.R435C or tagged wt *LMNA* plasmids. After treatment of the transfected cells with hydrogen peroxide, we observed multiple 53BP1 foci at DNA damage sites in nuclei expressing human wild-type LMNA, whereas in p.R435C-*LMNA* transfected nuclei a low amount of foci appeared at the same time of treatment (Fig.7). Thus, while LMNA p.R435C seems able to localize to its normal nuclear lamina region (mentioned above), its ability to recruit 53BP1 to damaged DNA sites appears to be impaired.

## DISCUSSION

Progeroid phenotypes resembling Restrictive Dermopathy (RD) have been linked to primary or, more frequently, to secondary laminopathies [15]. In primary age-related laminopathies with dominant inheritance, accumulation of mutated prelamin A precursors triggers toxic nuclear effects in a dominant-negative manner resulting in premature aging, more similar to HGPS. In the case of homozygous *ZMPSTE24* null mutations, which cause a complete loss of mature lamin A protein, the clinical course of the disease is fatal, foudroyant, and associated with prenatal or neonatal death. Individuals harbouring heterozygous *ZMPSTE24* mutations are clinically inconspicuous, as the amount of normal ZMPSTE24 is adequate to process prelamin A to mature lamin A sufficiently [7, 9]. Intriguingly, a case of atypical progeria associated with heterozygous compound mutations in *ZMPSTE24* has been described that showed mature lamin A decrease [8].

In the present study, we identified a fatal RD-like disease as a distinct primary progeroid laminopathy linked to the homozygous pathogenic *LMNA* mutation c.1303C>T (p.R435C). Apparently the heterozygous mutation occurs at a low frequency in the normal population. So far the heterozygous mutation *LMNA* c.1303C>T (p.R435C) has been associated in one reported case to dilated cardiomyopathy [14]. We therefore performed cardiologic assessment of four of the five heterozygous family members related to the fatality. Except for one individual with mild signs of hypertensive cardiac disease (most likely age related), none of the individuals analysed showed any clinical or echocardiographic evidence of functional or structural cardiac impairment. Additionally, in another study including three heterozygous individuals, no cardiac abnormalities have been reported [13]. Thus, it seems unlikely that the heterozygous LMNA p.R435C mutation alone can be associated to a pathogenic effect. Perhaps additional risk factors are necessary to trigger a cardiomyopathy.

Considering the low frequency of LMNA p.R435C in the normal population, the occurrence of homozygous individuals is extremely rare; assuming Hardy-Weinberg equilibrium, the likelihood was 5×10^−8^. However, homozygosity for very rare alleles often occurs on a consanguineous background. This has indeed been found in a Polish family, where the only homozygous LMNA p.R435C has been reported so far [13]. Additionally, as shown for the first time by this study, partial uniparental disomy can result in rare homozygous *LMNA* mutations.

In contrast to the heterozygous *LMNA* p.R435C mutation, which expresses a rather mild clinical picture if any, the patient harboring the homozygous mutation presents clinically with a severe progeroid syndrome including severe skin abnormalities as pathognomonic symptoms. Follow-up of the clinical course of the patient for 11 months showed that the phenotype represents a distinct primary progeroid laminopathy, harboring clinical and morphologic overlapping features of RD, HGPS and MAD. The fact that the patient was nearly inconspicuous at birth resembles HGPS; absence of hairiness is typical for RD and HGPS. Skin changes and loss of claviculae have been reported in all three syndromes mentioned, but particularly the generalized tight and thick skin with epidermal thickening, reduction of the rete ridges and rarefication of skin appendages, combined with missing hairiness are pathognomonic features of RD. In contrast to classic RD, the patient had a thickened dermis and subcutaneous lipodystrophy. However, the predominant phenotype of the patient was conditioned by a dramatic progression of the skin abnormalities resulting in microstomia, flexion contractures of the extremities, rocker bottom feet, combined with facial abnormalities like hypolastic nose and micrognathia, all in all resembling RD. Whereas the typical RD patient is diagnosed neonatally in a pre-final stage of disease, similarly the patient developed a “dermal exoskeleton” causing disturbed temperature regulation and global respiratory insufficiency leading to death. In summary, the clinical course of the reported patient appears to one as an accelerated HGPS finally ending up in a delayed RD with overlapping clinical features of MAD.

The patient's progressive skin changes were dramatic and reflected the beginning of a vicious cycle leading to encasement of the patient, loss of mobility, severe growth and developmental delay, muscular hypotension, respiratory impairment and finally death from pneumonia, which is common in RD patients. X-ray analyses at two time points indicated that a continuous degeneration of initially normal appearing bone tissue (claviculae, humeri) occurred. Thus, deformation and absence of bone tissue as a leading symptom of progeroid phenotypes may not be a developmental or differentiation error, but may rather illustrate a symptom of disease progression as it occurs at an advanced stage of the disease.

There are ongoing discussions about the molecular mechanisms of premature aging syndromes including laminopathies and which aspects might resemble physiological human aging. Increased DNA damage, defects in DNA repair mechanisms and shortening of telomeres are characteristics of normal aging. Highlighting one link to the family of laminopathies, telomeres may directly be damaged by Progerin in HGPS (although involvement of telomeres has not been investigated in the present study). Likewise persistent DNA damage is one attribute of other premature aging diseases, even if not all *LMNA* mutations result in DNA damage and genome instability [16]. However, experimental downregulation of *LMNA* can lead to accelerated nuclear senescence and apoptosis [17], and altered lamin A function has even been described as an indicator of senescence during physiological aging process [18].

Immuno-histological analyses of tissue samples taken at the beginning and the end of the disease course allowed for the first time a follow-up of functional changes associated with this homozygous *LMNA* mutation, and thus provided insight into molecular pathogenic processes of progeroid primary laminopathies. We observed a progressive decrease in lamin A levels in the skin of our patient during the worsening of the clinical phenotype (Fig.5). The progression resembles that found in homozygous lamin A knockout mice [19], which were indistinguishable from heterozygous or wild type at birth, but then showed severely retarded postnatal growth and markedly reduced life span. In contrast, the heterozygotes developed apparently normally and did not exhibit any premature mortality when compared to wild type mice. Interestingly, cells from the only *LMNA*-null patient so far reported [20, 21]showed mislocalization of pRb and early cell cycle arrest possibly due to interference of a cell cycle checkpoint [22].

Activated DNA damage response (DDR) has been discussed as another indicator of replicative senescence and accelerated aging [23]. DDR is thought to play a role in the repair of short-range DNA dsb by binding to the breaks, inhibiting end-resection and facilitating the recruitment of the non-homologous end-joining DNA repair machinery.

It was intriguing that in parallel with the progressive loss of lamin A high levels of DNA dsb occurred in our patient, indicative of increasing DNA damage (Fig.5). It has recently been shown that among other downstream targets p53 signaling pathway can be altered by LMNA dysfunction [24]. Moreover, lamin A is involved in the stabilization of P53 binding protein 1 (53BP1), a component of the DDR pathway [12].

A recent report suggested that the loss of 53BP1 could be responsible for the DNA repair deficiencies observed in LMNA-K.O. cells [25]. Additionally, depletion of lamin A in cultured primary cells has been shown to trigger a senescent cell cycle arrest [26]. Since we found (Fig.6) that following oxidative stress-induced DNA damage very few 53BP1 foci occurred in LMNA R435C transfected cells, it is possible that the DNA repair is delayed or impaired due to the lamin A mutation. It should be pointed out that our results could be in part hampered by an overexpression effect. In fact, a synergistic effect derived from the *LMNA* mutation combined with protein deficiency appears likely in vivo. For instance, we might expect a reduced importation of 53BP1 into nuclei, which was not observed in LMNA p.R435C transfected fibroblasts. One could speculate that because the aberrant, non-functional, mutant lamin A has a functionally negative effect and likely abnormal conformation, it therefore could also consequently undergo greater degradation than wt-lamin A. The phosphorylation of the histone H2AX at Serine 139 (γH2AX) is the earliest chromatin modification after exposure to ionizing radiation [27] and is thought to be involved in the stable accumulation of 53BP1. But 53BP1 can still be recruited to dsb sites in H2AX null cells, which show only mild defects in DNA damage checkpoint control and DNA repair [28]. Therefore it seems likely that the DNA damage in our patient occurs due to a mis-localization of 53BP1. Unfortunately, we could not use living cells from the patient to test such hypotheses. However, we failed to observe any effect of R435C lamin A in transfected HEK293 embryonic cells, which express very low levels of endogenous lamin A (not shown). This finding argues in favor of a specific role of lamin A in recruiting 53BP1 at DNA damage sites in post-natal cells.

Involvement of this *LMNA* mutation in DNA damage may be of clinical importance for future patients, as it has been observed that DDR may activate mTOR signaling and in turn drive cellular senescence. Suppression of cellular senescence by using the mTOR-inhibitor Rapamycin has been discussed as an innovative therapeutic approach in the clinical treatment of laminopathies (reviewed in [23]). However, further investigations are needed to assess a possible clinical benefit of patients harboring the reported or other *LMNA* mutations accompanied by increased DNA damage.

Our data support the idea that the loss-of-Lamin A itself and/or its function in LMNA p.R435C homozygous individuals leads to a loss of DNA-damage-prevention, accompanied by accumulation of double strand breaks, being reflected by progressive degeneration of tissues, osteolysis and cutaneous rigidity as seen in the presented patient. In conclusion, our clinical and functional findings provide a link between the functional loss of lamin A via disturbed DNA repair pathways and degeneration of cells that results in a progeroid phenotype as observed in the presented case. Previously, increased genomic instability has been observed in other progeroid laminopathies including HGPS as well as RD, and is thought to be associated with prelamin A or progerin accumulation [10, 11]. In contrast, our case provides evidence that increased DNA damage/genomic instability in progeroid laminopathies is due to the lack of mature, functional lamin A rather than the abnormal accumulation of prelamin A forms.

## MATERIALS AND METHODS

### Patients and family members

This Research has been approved by the clinical ethics committee / internal review board of the University Children's Hospital Leipzig.

Informed consent has been obtained for the patient and all individuals studied in this report (see Results section).

### DNA extraction

Genomic DNA of the patient was extracted a blood sample or from paraffin embedded tissue that had been obtained at autopsy, using QIAamp DNA-Minikit^®^ according to the manufacturer's protocol. Acceptable quality genomic DNA was confirmed by agarose-gel analysis under standard conditions. Genomic DNA of investigated family members was isolated from peripheral blood lympho-cytes using QIAamp-DNA-Minikit^®^.

### PCR and Sanger sequencing

Primer pairs for all the coding exons and flanking intronic sequences of *LMNA* (ENSG00000160789, ENST00000368300) and *FACE1* (ENSG00000084073, ENST00000372759) are available in Table S2. Microsatellite markers were amplified using the primers available in Table S1. To standardize the sequencing reaction, all *LMNA* and *FACE1* primers were tagged with a M13-tail (forward: 5'-GTAAAACGACGGCCAGT-3' reverse: 5'-CAGGAA ACAGCTATGAC-3'). Amplifications were performed in 25 μl volumes using Amplikon-Taq Polymerase (Biomol) under the following thermal conditions: initial denaturation at 94° for 5 min followed by 30 cycles of denaturation (94°C for 15 sec), annealing at the appropriate temperature for 15 sec (see Tables S1 and S2) and elongation (72°C for 1 min). A final elongation (72°C for 7 min) preceded a 4°C cooling step. To amplify exon 9 of the *FACE1* gene, a proof reading (high fidelity) Pfu polymerase (Fermentas) was used. In agarose gel electrophoresis, 3 μl of the PCR product were analysed using a 1,5% agarose-gel to test the PCR efficiency. PCR products were used to identify changes by direct Sanger sequencing. Excess dNTPs and primers were removed using ExoSAP-IT^®^ (Affymetrix). Sequencing reactions were performed using ABI BigDye^®^ Terminator v3.1 Cycle Sequencing Kit with addition of 5% DMSO to the reaction mix. M13-oligonucleotides were used as sequencing primers for *LMNA* and *FACE1* analysis, for the microsatellite analysis the PCR primers were used as sequencing primers. The reactions were analysed on a 3130xl GA DNA Sequencer (Applied Biosystems) according to the manufacturer's instructions. All DNA variations identified were validated using a second independent DNA sample.

### Multiplex Ligation-dependent Probe Amplification (MLPA)

MLPA analysis was performed using the Salsa MLPA P048-B1 LMNA probemix from MRC Holland according to the manufacturer's instructions. The reactions were analysed on an ABI PRISM^®^ 310 DNA Sequencer (Applied Biosystems) according to the manufacturer's instructions.

### Histochemistry and Immunostaining

Paraffin embedded tissue samples were enclosed at the Department of Dermatology and the Institute of Pathology, University of Leipzig at patient's age of four months, and when autopsy was performed at patient's age of 11 months, respectively. The paraffin embedded blocks were sectioned in 3-10μm slices and mounted on adhesion microscope glass slides (SuperFrost plus, Menzel J1800AMNZ). The sections were deparaffinised in xylene for 3 minutes and rehydrated through a series of graded ethanol (100% for 3 min, 96% for 3 min, 70% for 3 min, water for 3 minutes). For antigen demasking, slides were cooked for 20 minutes in Target Retrieval Buffer pH 9,0 (Dako S2367) using a steamer.

For lamin A staining, endogenous peroxidases were blocked with Dual block (Dako S2003) for 10 minutes. After three washes in Tris Buffered Saline (TBS) for 3 minutes, control (no primary antibody) and experimental slides were incubated for 50 minutes at room temperature, respectively, in Dual Block (Dako S2003) alone, or with lamin A/C antibody (cell signalling 2032) at 1:50 dilution.

For H2AX stain, endogenous peroxidases were blocked with 3% hydrogen peroxide for 10 minutes. After three washes in Tris Buffered Saline (TBS) for 3 min., slides were incubated in 5% Serum Block Goat (Dako X0907) for 30 min at room temperature. Control (no primary antibody) and experimental slides were incubated overnight at 4°C followed by 30 minutes at room temperature, respectively, in 5% Serum block goat alone (Dako X0907), or with H2AX antibody (abcam ab2893) at 1:500 dilution.

After washing in TBS buffer, sections were incubated for 30 minutes at room temperature with horseradish peroxidase (HRP) labelled goat anti rabbit secondary antibody (Dako P0448), using EnVision+ System-HRP (DAB) Kit (Dako K4011). After washing in TBS, 3,3'-Diaminobenzidine (DAB) solution was applied for five minutes. Exposure times were synchronized so that all tissues samples within an antibody group were exposed to DAB for the same time. All stains were counterstained with Mayers Hämalaun (Roth T865.1) for approximately 1 minute and washed in tap water for ten minutes, dehydrated in ethanol, cleared in xylene and mounted with Entellan (Merck 1.07961.0100). Images were obtained with 20× magnification using a Keyence Biozero microscope and digital camera. Exposure times were kept constant for all samples.

### Plasmid constructs and site-directed mutagenesis

The p.R435C mutation was introduced in the pLEICS-23 plasmid, which was carrying a mCherry-tagged *LMNA* insert, by the PROTEX cloning service of the Department of Biochemistry, University of Leicester.

### Cell culture and transfection

Human fibroblast cultures were obtained from skin biopsies of consenting patients undergoing orthopaedic surgery for traumas. Cell cultures were established by mechanical and enzymatic methods and routinely sub-cultured in D-MEM plus 20% FCS and antibiotics. Fibroblasts were transfected with plasmids encoding mCherry-tagged wild-type lamin A or R435C lamin A. Transfection was performed using an AMAXA electroporator (Amaxa, Lonza Group, Basel, Switzerland), according to the manufacturer's instructions. After transfection, cells were incubated for 42 hours and treated with 100μM hydrogen peroxide for 5 hours.

### Immunolabeling of cells and tissues

Fibroblasts grown on coverslips were fixed in 100% methanol for 7 min, saturated for 25 min with 4% BSA and subjected to immunofluorescence staining using a monoclonal anti-53BP1 antibody (Cell Signaling) applied at 1:30 dilution. Skin biopsies were fixed in 10% formaldehyde and embedded in paraffin according to standard procedures. Paraffin-embedded sections (7 μm) were immunolabeled with anti-prelamin A 1188-1 antibody (Diatheva), which was applied overnight at 4°C at 1:50 dilution. Bound antibody was detected with a horseradish peroxidase-conjugated anti-rabbit Ig, using diaminobenzidine (DAB) as a substrate. Samples were counterstained with hematoxylin. A Nikon E600 epifluorescence and bright field microscope and a Nikon oil-immersion objective (100x magnification) were used. Images were processed using Adobe Photoshop 7.0 software.

### Antibodies

anti-lamin A/C antibody (Cell Signalling Technologies #2032)

anti-H2AX antibody (abcam #2893)

anti-prelamin A (1188-1)

anti-53BP1 antibody (Cell SignalingTechnologies #4937).

## SUPPLEMENTAL TABLES AND FIGURES


