# Supplementary material for: Progeroid laminopathy with restrictive dermopathy-like features caused by an isodisomic LMNA mutation p.R435C
Source: Aging (Albany NY). 2013 Jun 19;5(6):445–59. doi: 10.18632/aging.100566 (PMC3824411; doi:10.18632/aging.100566)
Supplement: Supplementary file 1 [file aging-05-445-s001.pdf]

SUPPLEMENTAL TABLES

Table S1. Primer sequences and annealing temperatures for genomic amplification of *LMNA* and *FACE1* exons

Exon	Orientation	Primer sequence (5'-3')	Amplicon size (bp)	Annealing temperature (°C)
LMNA E1a	Forward	GGGACTGCCCCTTTAAGAGT	500	62
	Reverse	GACCACCTCTTCAGACTCGG		
LMNA E1b	Forward	ACATCGACCGTGTGCGCTC	268	62
	Reverse	CCTCTCCACTCCCCGCCA		
LMNA E2	Forward	GATGCCCTCTCCTGGTAAT	399	60
	Reverse	GCTCTGAAATCAGGTGACAGG		
LMNA E3	Forward	TTCTTGTGTTCTGTGACCCCTT	232	60
	Reverse	CCCAAGTCTGTCATCACCCA		
LMNA E4	Forward	TAAAGTGGGGCTGGTAGTGG	319	62
	Reverse	GTGAGGGAACCAATCGAGAG		
LMNA E5	Forward	CCTCCACCCCTCCCAGTCAC	231	62
	Reverse	TGCATCCGCCCCAGACTCTA		
LMNA E6	Forward	CAAACCCTCCCACCCCCC	299	62
	Reverse	CCAGTTGCCGGGCCAGAG		
LMNA E7	Forward	CCCCACTTGGTCTCCCTCTCC	292	62
	Reverse	CCCTGATGCAGCTGTATCCCC		
LMNA E8	Forward	TGGGCCTTTGAGCAAGATAC	257	60
	Reverse	GAAAAGGACACTTACCCCAGC		
LMNA E9	Forward	CAGGTGGTGACGGTGAGTG	285	62
	Reverse	CAGCTGGCTCCGATGTTG		
LMNA E10	Forward	GCCACAAGAAAAGTTGCAGG	320	62
	Reverse	CAGGCCAGCGAGTAAAGTTC		
LMNA E11	Forward	TGGTCAGTCCCAGACTCGCC	358	60
	Reverse	CGCCTGCAGGATTTGGAGA		
LMNA E12	Forward	TGAGGGATGGGGGAGATGCT	196	62
	Reverse	GGGTGGGCATGAGGTGAGGA		
FACE1 E1	Forward	GGGCTGGGGCTTTTCCTGTAG	472	55
	Reverse	GGACCACAAAGACGAGACTGG		
FACE1 E2	Forward	TGGCAAGCTATAAACCATTCG	289	55
	Reverse	GAAAATGAAAACAACCAGAC		
FACE1 E3	Forward	CCGTACTGGCCTCTTTTGTT	273	55
	Reverse	GAAAGCCTGCCAAGCTAAAA		
FACE1 E4	Forward	TTGATTTGTTTGCCAGTAGTTCA	235	55
	Reverse	CAGGACAAAAGCACAGAAGTTTT		
FACE1 E5	Forward	CCAGTTTCTCAGTTTCTTGTGG	244	55
	Reverse	TCTCACCAAGGAACTTTTGC		
FACE1 E6	Forward	GGGCCTGGGAATACCAGAGCAAG	490	55
	Reverse	AGCCACCAGTTTCTATCCCTGGC		
FACE1 E7	Forward	CTCCAAAGGACCCCAAACTT	310	55
	Reverse	TTTTGAGTTGTCACAGGAACTG		
FACE1 E8	Forward	AATCTATGAAGGGCTATTACTG	278	55
	Reverse	CTTGGCCTCTCTTATATGAC		
FACE1 E9	Forward	TGATCCCATAGTGAAATCAGCTT	256	55
	Reverse	GATTTGAAGCAGGCAAGAGC		
FACE1 E10	Forward	TACAGTCTCAGCTCATGGAAC	357	55
	Reverse	TGCTGCCAGGACAGAAATAA		

Table S2. Primer sequences of microsatellite markers used for the analysis of uniparental disomy on chromosome 1

Marker	Position	Orientation	Primer sequence (5'-3')	Annealing temperature (°C)
D1S2660	1p36	Forward	CACACATGCACATGCAC	50
		Reverse	AGTGACACCAGCAGGG	
D1S2652	1p32	Forward	GCAGGTGTGATGCCAGG	50
		Reverse	TACGGCTGATTGGGAGAAC	
D1S500	1p31	Forward	GTAATGTCACTGGCATGGA	50
		Reverse	CTCTGATACGCCAAGTGCT	
D1S2726	1p13	Forward	CCACAAGTTGCAGGGTT	50
		Reverse	CTGGATGGATGCTCAAATAC	
D1S498	1q21.3	Forward	TTGCTGAAGGGACATAGTG	50
		Reverse	TGCTGGGTTATATCCAATATC	
D1S3020	1q21.3	Forward	TGGTGTTTGGTTACATGGAT	50
		Reverse	GTGAAGGCAACATGTATCGT	
D1S305	1q21.3	Forward	CCAGNCTCGGTATGTTTTTACTA	50
		Reverse	CTGAAACCTCTGTCCAAGCC	
D1S2140	1q22	Forward	GCTGAAAAGACACTTCAGTGG	50
		Reverse	ATGGTATGAACCTGGAGGTG	
D1S1653	1q23.1	Forward	GGAAAGCCTGTAGGAAGAGG	50
		Reverse	CCTGGATGACAGAGTGCTCT	
D1S2369	1q23.1	Forward	ACATCCATCCTTAATATTTTGGC	50
		Reverse	GCATTTCTGACACTCATGACTTG	
D1S484	1q23.3	Forward	AGTGATGAGGGCCTCTATTT	50
		Reverse	AGCTTCTGCCAACTATGTGC	
D1S210	1q24	Forward	CCTCAGTTCATTCCCCATAA	50
		Reverse	AGCTGAATCTCACCCAATAACTA	
D1S249	1q32	Forward	TGGCATGTCTTTGAAGGAAT	50
		Reverse	TGGTTGTAGATGAGACTGGC	
D1S423	1q44	Forward	GGGCAACAAGAATGAAACTC	50
		Reverse	GGCCTAAGATTGCTGTGAAG	

SUPPLEMENTAL FIGURES

Change		Exon	Reference (Ensembl)	Frequency (Reference population)
cDNA	Amino Acid			
c.861T>C	p.A287A	5	rs17847240	16%
c.1157+16G>A	---	6	rs57888699	16%
**c.1303C>T**	**p.R435C**	**7**	**rs150840924**	**0,023%**
c.1338T>C	p.D446D	7	rs17847243	21%
c.1489-41C>T	---	8/9	rs17847245	15%

Supplementary Figure S1. DNA changes found by Sanger sequencing of the coding region of the *LMNA* gene (ENSG00000160789, ENST00000368300), rs numbers and frequencies of the changes found in a European reference population (http://ensembl.genomics.org.cn:8058/index.html).

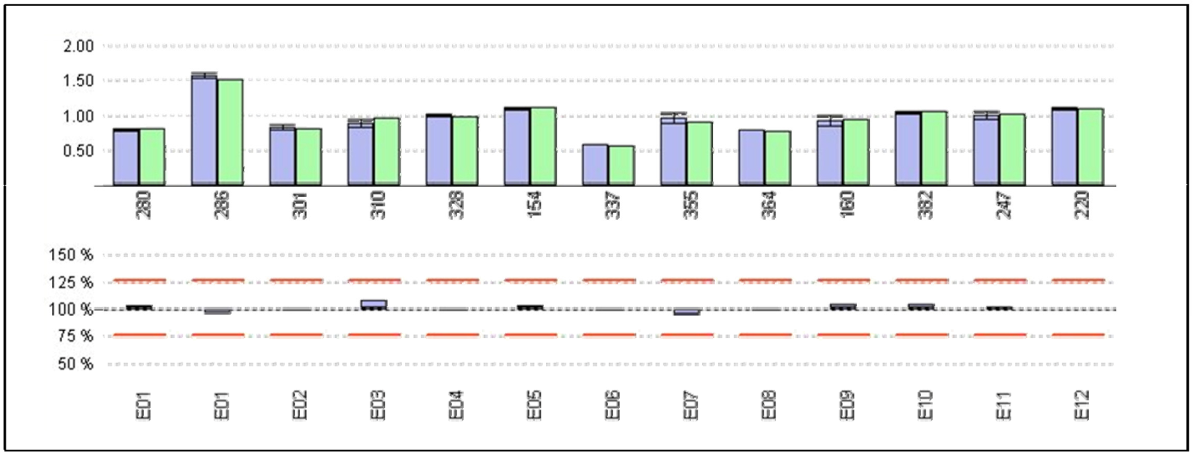

Supplementary Figure S2. Multiplex Ligation-dependent Probe Amplification (MLPA) analysis for the *LMNA* gene performed on DNA of the index patient. There are two copies for all 12 exons of the *LMNA* gene.

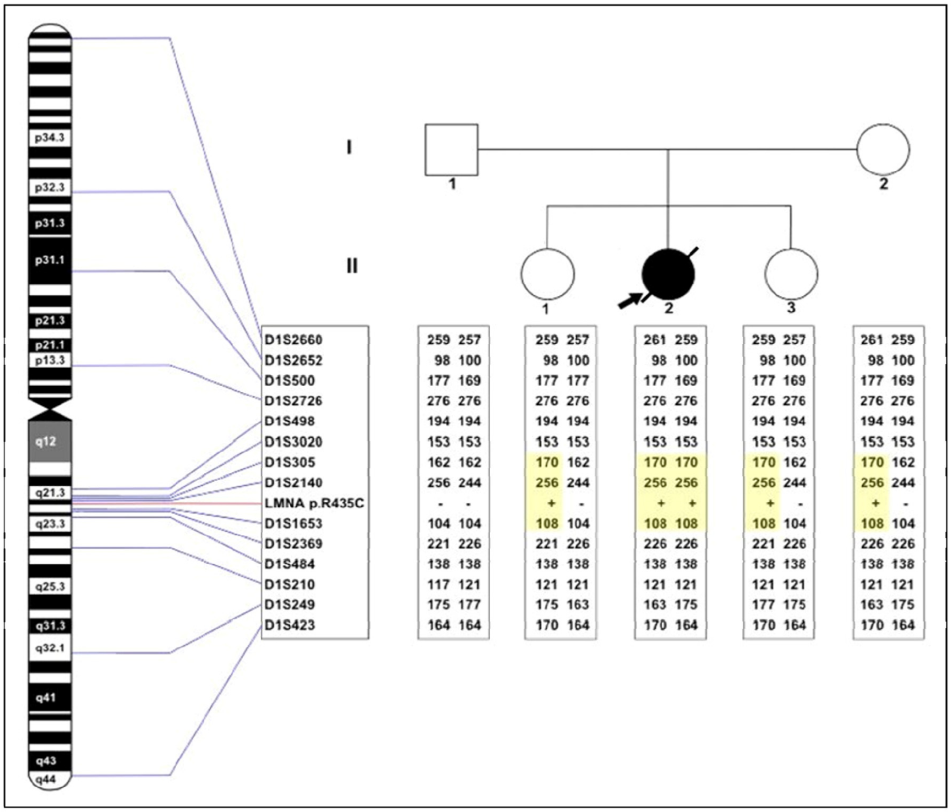

Supplementary Figure S3. Microsatellite marker analysis on chromosome 1 showing the position of the chosen markers and their length in base pairs for the index patient, both parents and both siblings. The mutant *LMNA* allele is marked as +, the wild type allele as -. The region marked in yellow is evident for uniparental disomy.

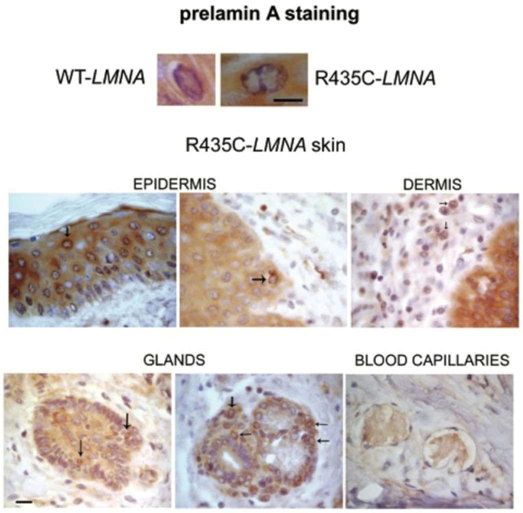

Supplementary Figure S4. Prelamin A-staining of skin samples taken at age of 11 months. Only a few nuclei in the patient's skin were positive for prelamin A. This is confirming that the processing of prelamin A is not affected.
